# Cor Triatriatum Sinister With a Patent Foramen Ovale Presenting With Embolic Stroke

**DOI:** 10.1016/j.jaccas.2025.103549

**Published:** 2025-06-04

**Authors:** Ryo Sakuma, Mary Carter Denny, Yue-Hin Loke, Michael Slack, Seiji Ito

**Affiliations:** a US Naval Hospital Yokosuka, Kanagawa, Japan; b Vascular Neurology, MedStar Health Georgetown University Medical Center, Washington DC, USA; c Cardiology, Children’s National Hospital, George Washington University, Washington, DC, USA; d Cardiology, MedStar Washington Hospital Center, Georgetown University, Washington DC, USA; e Pediatrics, University of Maryland School of Medicine, Baltimore, Maryland, USA

**Keywords:** Cor triatriatum sinister, embolic stroke

## Abstract

**Images:**

Cor triatriatum sinister/echocardiogram and cardiac computed tomography.

**Case Summary:**

The patient was a 21-year-old woman with an embolic stroke who was found to have an unobstructed Cor triatriatum sinister (CTS) and patent foramen ovale and underwent a patent foramen ovale device closure and subsequent anticoagulation therapy. Case reports of CTS associated with stroke have been previously published, and CTS is recognized as a risk factor for an embolic stroke; however, the mechanism of embolic stroke is unclear.

**Take-Home Message:**

Further study is warranted to understand the mechanism and role of CTS for embolic stroke and optimal therapeutic options to reduce the risk of recurrent stroke.

## Graphical Abstract

**Figure:**
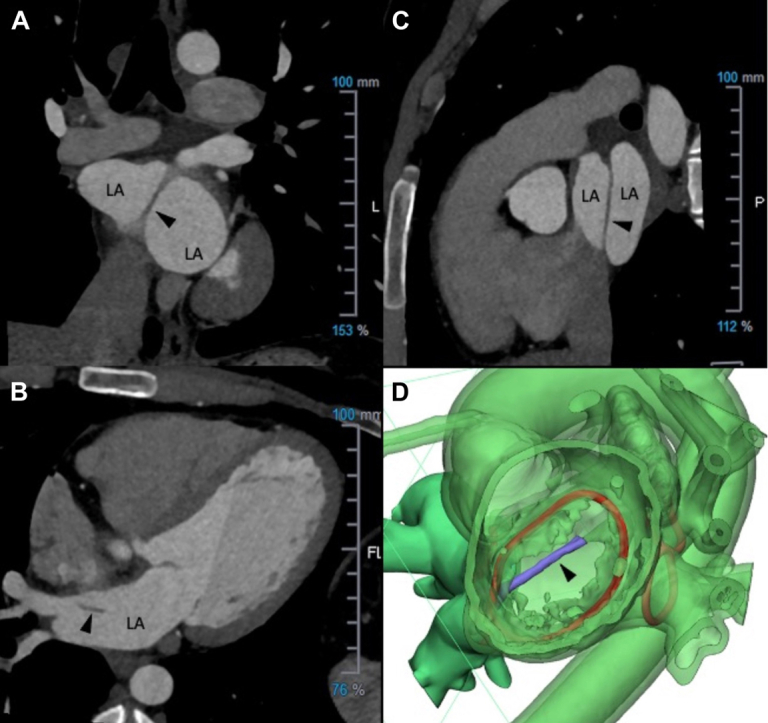

## Case Summary

**Figure 1:**
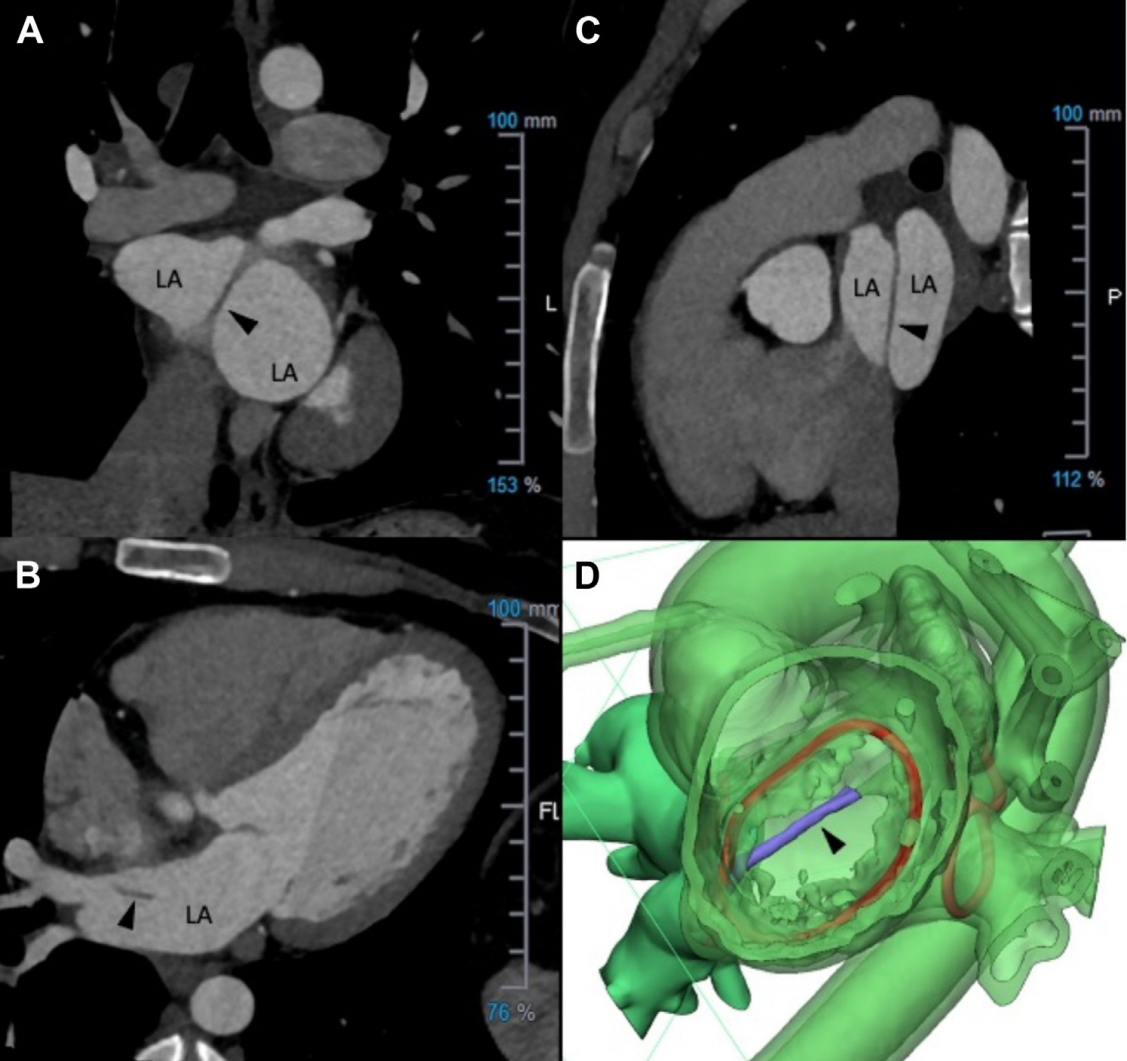
Cor Triatriatum Sinister Cardiac computed tomography image in coronal plane (A), axial plane (B), and sagittal plane (C). Apical basal view of 3-dimensional digital cardiac image of Cor triatriatum sinister (CTS) (D). Arrowheads point to a band of CTS tissue. In D, the CTS is shown in purple, and mitral valve annulus is shown in the red circle in center. LA = left atrium.

A 21-year-old woman with a history of oral contraceptive use presented to the emergency department with a headache, inability to get up from bed, aphasia, and right-sided numbness. An emergent head computed tomography (CT) and magnetic resonance imaging revealed an acute left middle cerebral artery infarct with a thrombus at the distal left middle cerebral artery. The patient underwent a cerebral angiogram with thrombectomy. Her oral contraceptive was discontinued, and she was started on aspirin 81 mg daily with gradual improvement of neurologic deficits over 3 months. The evaluation for stroke etiology revealed no thrombophilia; no vascular anomaly on CT angiogram of the chest, abdomen, and pelvis; and no atrial fibrillation on extended cardiac rhythm monitoring. Transthoracic and transesophageal echocardiogram confirmed a patent foramen ovale (PFO) with grade Ⅱ interatrial shunting. Her biventricular systolic function was normal, and no intracardiac thrombus was seen. However, a prominent but nonobstructive band of tissue in the left atrium extending from the Coumadin ridge to the inferior portion of the atrial septum was visualized, suggesting an underlying Cor triatriatum sinister (CTS) (Video 1). A cardiac CT also demonstrated the CTS (Figure 1, Video 2). The patient’s risk of paradoxical embolism score of 7 estimated a high chance the stroke was due to PFO. After comprehensive discussion, the patient underwent PFO closure (Amplatzer Talisman PFO Occluder 30/25; Abbott). Furthermore, oral anticoagulation therapy and surgical resection of CTS were also considered as additional therapeutic options. Through shared decision-making, oral anticoagulation with apixaban 5 mg twice daily was started, but surgical resection was not pursued. At 18 months after the initial presentation, she remained well with no stroke recurrence; however, apixaban was switched to rivaroxaban for ease of once-daily dosing. Cases of CTS associated with stroke or recurrent strokes have been reported sporadically, including cases without PFO. The exact mechanism of embolic stroke associated with CTS remains unknown. In the setting of PFO-associated stroke, guidelines from the Society for Cardiovascular Angiography and Interventions support the closure of PFO rather than anticoagulation or antiplatelet therapy alone.^1^ Regarding CTS, the current adult congenital heart disease guideline supports surgical resection of the CTS if symptoms are attributable to the CTS obstruction, or a substantial gradient exists across the CTS.^2^ However, whether a CTS resection reduces the risk of recurrent stroke is unknown. A recent clinical consensus statement on embolic strokes of undetermined source emphasized assessing overall thromboembolic risk rather than presuming actual embolic source. CTS was noted as one of the features of left atrial disease associated with thromboembolic risk.^3^ Further study is warranted to understand the mechanism and role of CTS for embolic stroke and optimal therapeutic options to reduce the risk of recurrent stroke.

**Take-Home Messages**

• The mechanism of embolic stroke in CTS remains unclear.
• Further study is warranted to understand the mechanism and role of CTS for embolic stroke and optimal therapeutic options to reduce the risk of recurrent stroke.

## Funding Support and Author Disclosures

Dr Denny is on the Speakers Burearu for Abbott. Dr Slack is teaching proctor and consultant for Abbott. All other authors have reported that they have no relationships relevant to the contents of this paper to disclose.

## Appendix

Video 1 Transesophageal Echocardiogram Image of Cor Triatriatum Sinister Transesophageal echocardiogram image in 3-chamber view plane showing a band-like structure in the left atrium.

Video 2 Cardiac CT Images of CTS in Coronal and Axial Plane CT images showing a band-like tissue of CTS extending from the coumadin ridge to the inferior portion of the atrial septum, shown in coronal and axial planes.

## References

[1] Kavinsky C.J., Szerlip M., Goldsweig A.M. (2022). SCAI guidelines for the management of patent foramen ovale. J Soc Cardiovasc Angiogr Interv.

[2] Stout K.K., Daniels C.J., Aboulhosn J.A. (2019). 2018 AHA/ACC Guideline for the Management of Adults With Congenital Heart Disease: Executive Summary: A Report of the American College of Cardiology/American Heart Association Task Force on Clinical Practice Guidelines. J Am Coll Cardiol.

[3] Ntaios G., Baumgartner H., Doehner W. (2024). Embolic strokes of undetermined source: a clinical consensus statement of the ESC Council on Stroke, the European Association of Cardiovascular Imaging and the European Heart Rhythm Association of the ESC. Eur Heart J.

